# Inflammation, childhood trauma, and symptom dimensions in schizophrenia: a path-analysis study

**DOI:** 10.1016/j.bbih.2025.101060

**Published:** 2025-07-16

**Authors:** Francisca Silva, Andrei Szöke, Mohamed Lajnef, Myrtille André, Christelle Andrieu, Jihene Bouassida, Wahid Boukouaci, Fabrice Berna, Delphine Capdevielle, Maud Cléry, Isabelle Chéreau, Nathalie Coulon, Julia Clauss-Kobayashi, Eric Fakra, Jean-Michel Dorey, Caroline Dubertret, Guillaume Fond, Ophélia Godin, Tudi Goze, Marion Leboyer, Sylvain Leignier, Pierre-Michel Llorca, Jasmina Mallet, David Misdrahi, Nicolas Oriol, Romain Rey, Paul Roux, Benoit Schorr, Arnaud Tessier, Ryad Tamouza, Mathieu Urbach, Etienne Véry, Ching-Lien Wu, the FACE-SZ (FondaMental Academic Centers of Expertise - Schizophrenia) group, M. Andre, C. Andrieu-Haller, B. Aouizerate, F. Berna, O. Blanc, D. Capdevielle, M. Cléry, I. Chereau-Boudet, J. Clauss-Kobayashi, N. Coulon, R. Dassing, J.M. Dorey, C. Dubertret, A. Esselin, G. Fond, F. Gabayet, M. Jarroir, D. Lacelle, M. Leboyer, S. Leignier, P.M. Llorca, J. Mallet, E. Metairie, T. Michel, D. Misdrahi, C. Passerieux, B. Pignon, P. Peri, C. Portalier, R. Rey, C. Roman, B. Schorr, F. Schürhoff, A. Szöke, A. Tessier, M. Urbach, A. Zinetti-Bertschy, Baptiste Pignon, Franck Schürhoff

**Affiliations:** a FondaMental Foundation Créteil, F-94010, France; b Univ Paris-Est-Créteil (UPEC), AP-HP, Hôpitaux Universitaires « H. Mondor », DMU IMPACT, INSERM, IMRB, Translational Neuropsychiatry, Créteil, France; c General and University Psychiatry Department, Charles Perrens Hospital, F-33076, Bordeaux, France; d Univ. Bordeaux, CNRS, INCIA, UMR 5287, F-33000, Bordeaux, France; e University Hospitals of Strasbourg, Department of Psychiatry, France; f University of Strasbourg, Inserm U1114, Strasbourg, France; g CHU Clermont-Ferrand, Service of Psychiatry B, University of Clermont Auvergne, Clermont-Ferrand, France; h Grenoble Alpes University, Inserm U1216, CHU Grenoble Alpes, Grenoble Institute of Neurosciences, Grenoble, France; i Department of Psychiatry, University Hospital of Saint-Etienne, Saint-Etienne, France; j Le Vinatier Hospital, Schizophrenia Expert Centre, Bron, F-69500, France; k INSERM, U1028, France; l CNRS, UMR5292, France; m University Lyon 1, France; n Lyon Neuroscience Research Center, PSYR2 Team, Lyon, F-69000, France; o AP-HP, France; p Department of Psychiatry, Louis Mourier Hospital, Colombes, France; q Inserm UMR1266, Institute of Psychiatry and Neuroscience of Paris, University Paris Descartes, France; r Université Paris Diderot, Sorbonne Paris Cité, Faculté de médecine, France; s APHM, Service de Psychiatrie universitaire, Aix-Marseille université, Marseille, France; t Department of Psychiatry, Psychotherapies, Art-therapy, Toulouse University Hospital, France; u Equipe de Recherche sur les Rationalités Philosophiques et les Savoirs - EA3051, Université de Toulouse - Jean Jaurès, Toulouse, France; v Versailles Hospital, Department of Adult Psychiatry and Addictology, Centre Hospitalier de Versailles, Service universitaire de Psychiatrie d'adultes et d'addictologie, Le Chesnay, France; w Université Paris-Saclay, Université de Versailles Saint-Quentin-En-Yvelines, France; x DisAP, MOODS team, INSERM UMR1018, CESP, Villejuif, France; y Service Universitaire de Psychiatrie Adulte, CHU de Montpellier, France; z IGF, Univ. Montpellier, CNRS, INSERM, Montpellier, France; aa fondamental foundation creteil f, 94010, France; ab Psychiatry department, CHU d’Orléans, EPSM du Loiret, Orleans, France; ac Laboratoire Interdisciplinaire pour l’Innovation et la Recherche en Santé d’Orléans (LI2RSO), équipe B-CLINE, Université d’Orléans, Orleans, France

**Keywords:** Schizophrenia, Childhood trauma, Inflammation, Cytokines, Biomarkers

## Abstract

**Introduction:**

It has been postulated that immune dysregulations may establish a link between early risk factors, such as childhood trauma (CT), and the later development of schizophrenia. Schizophrenia and experience of childhood trauma (CT) have independently been associated with increased circulating levels of inflammatory biomarkers, while CT and inflammation have also been linked to particular clinical features of the disorder. We investigated whether increased levels of inflammatory biomarkers were underpinned by CT among subjects with schizophrenia, and whether this inflammation mediated the relationship between CT and symptom dimensions.

**Methods:**

451 subjects from the FACE-SZ (“FondaMental Academic Centres of Expertise for Schizophrenia”) cohort were included. Path-analysis was used to evaluate direct and indirect relationships between Childhood Trauma Questionnaire (CTQ) scores, serum concentrations of C-reactive protein (CRP), interleukin (IL)-6 and tumour necrosis factor (TNF)-α, and Positive And Negative Syndrome Scale of Schizophrenia (PANSS) scales.

**Results:**

Significant associations between CTQ and PANSS scales and between the former and inflammatory biomarkers were found. Notably, CRP was positively predicted by emotional abuse (β = 0.10) and physical neglect (β = 0.12), and negatively by emotional neglect while IL-6 (β = −0.18) was negatively predicted by sexual abuse (β = −0.05). No indirect associations between CT on PANSS through effects on the inflammatory biomarkers were found.

**Conclusions:**

Our study confirmed the association between CT and PANSS dimensions and identified novel links between CT and inflammation among patients with schizophrenia, highlighting the need for further elucidation of the complex relationships between early stress and chronic inflammation in psychiatry.

## 1 Introduction

Schizophrenia spectrum disorders are severe psychiatric illnesses typically characterized by positive psychotic symptoms (i.e. delusions or hallucinations), as well as negative, disorganised and cognitive symptoms, although presentations can be highly heterogeneous (Lieberman and First, 2018). Schizophrenia has a complex, multifactorial aetiology and a neurodevelopmental component. It is associated with important impairments in day-to-day functioning and with a significant increase in mortality risk due to both natural (e.g. cardio-metabolic complications) and external (e.g. suicide) causes (Harvey et al., 2019; Laursen et al., 2012; Rutter, 2013).

The field of immuno-psychiatry has made significant progress in temporally integrating the effects of genetic and environmental risk factors on immune-brain interactions, aiming to explain the development of psychiatric disorders and the physical comorbidities frequently associated with early mortality. It is hypothesized that the activation of immune mechanisms in response to early-life environmental stressors, particularly in individuals with genetic vulnerability, underlies the neurobiological and clinical characteristics of psychotic disorders (Leboyer et al., 2016; Müller, 2018).

*Accordingly, there is abundant evidence for immune dysfunctions at different system levels in subjects with schizophrenia, namely, greater serum concentrations of acute phase reactant (*e.g.*, C-reactive protein or CRP) and of pro-inflammatory cytokines, such as interleukin (IL)-1β, IL-6, and tumour necrosis factor (TNF)-α, when compared to controls (*Ermakov et al., 2022; Fernandes et al., 2016; Goldsmith et al., 2016*)*. Abnormal inflammatory profiles in subjects with schizophrenia have been associated with poorer cognitive performance, metabolic syndrome and resistance to antipsychotic treatment (Bulzacka et al., 2016; Foiselle et al., 2022; Leboyer et al., 2021; Patlola et al., 2023). Furthermore, increases in specific inflammatory molecules have been related to particular clinical symptom dimensions, for instance, higher CRP levels were associated with positive symptoms and a link between CRP and TNF-α and negative symptoms has also been observed (Fernandes et al., 2016; Goldsmith and Rapaport, 2020). It has been envisaged that immune dysregulations may be the missing link between early risk factors for schizophrenia and later development of clinical symptoms and comorbid physical disorders (Danese and Baldwin, 2017; Leboyer et al., 2016).

Childhood trauma (CT) is known to increase both the risk of schizophrenia and severe evolution of the disease (Bailey et al., 2018; Baudin et al., 2016; Varese et al., 2012). Additionally, among subjects with schizophrenia, specific relationships between childhood trauma questionnaire (CTQ) subscales and clinical dimensions have been found, for instance, between sexual abuse and positive symptoms, and between physical neglect and physical abuse and negative and depressive symptoms (Bailey et al., 2018; Pignon et al., 2019; Sitko et al., 2014).

*Importantly, CT also appears to be associated to increased peripheral inflammation, as suggested by several studies and meta-analyses encompassing different clinical and general population samples. The inflammatory biomarkers most frequently associated with CT are CRP, IL-1β, IL-6, and TNF-α, parameters which have also been found to be elevated in schizophrenia (*Baumeister et al., 2016; Coelho et al., 2014; Danese et al., 2007; Ermakov et al., 2022; Fernandes et al., 2016; Goldsmith et al., 2016*)*. One meta-analysis has contested the existence of a significant relationship between some of these biomarkers and CT in general but attributed the significant associations found in some studies to particular trauma subtypes (Brown et al., 2021). Overall, these findings suggest that history of CT could be a significant predictor of the pro-inflammatory state seen in schizophrenia subjects, and could indicate a temporal relationship between CT, inflammation and clinical symptoms of schizophrenia.

The possible role of peripheral inflammation as a mediator between early life stress and psychopathology has been conjectured, although the evidence remains limited to date (Danese and Baldwin, 2017). The modest amount of research investigating a three-way relationship between CT, peripheral chronic inflammation and schizophrenia generally appears to support this hypothesis. Namely, serum levels of IL-6, TNF-α and CRP, as well as CTQ scores, are significantly higher in schizophrenia groups when compared with controls, and also higher in subjects with schizophrenia reporting a history of CT than in those who do not (Dennison et al., 2012; Quidé et al., 2019). *In their sample of individuals with schizophrenia,* Quidé et al. (2019) *additionally found that certain CT types significantly predicted the increased levels of specific inflammatory parameters, namely sexual abuse was positively associated with CRP levels*. Chase et al. (2019) also found a continuous positive association between ratings of adverse childhood experiences, IL-6 mRNA levels and the severity of positive symptoms in schizophrenia subjects. However, these results were based on exploratory studies with limited sample sizes and inconsistent methodologies. More data from larger cohorts are warranted to confirm these findings.

Therefore, our study aimed to investigate the relationship between different types of CT and relevant peripheral biomarkers of inflammation, and in turn between these biomarkers and clinical dimensions of the disorder, in a large, multi-centre French schizophrenia cohort. We hypothesized that: (i) CTQ scores would have a positive association with levels of the inflammatory parameters and with clinical symptoms scores on the Positive And Negative Syndrome Scale of Schizophrenia (PANSS) dimensions; and (ii) levels of the inflammatory parameters would have a positive association with scores on the PANSS dimensions. The presence of both direct and indirect (through an intermediary impact on the inflammatory parameters) effects of CT on PANSS dimensions, would support a mediatory role of peripheral inflammation in the link between CT and clinical dimensions of schizophrenia.

## 2 Methods

### 2.1 Subjects

*The study sample was drawn from patients who were evaluated in hospitals belonging to the network of French Schizophrenia Expert Centres.* The FACE-SZ (“*FondaMental Academic Centres of Expertise for Schizophrenia*”) cohort has been described in detail elsewhere (Schürhoff et al., 2015). Subjects meeting the DSM-5 criteria for the diagnosis of schizophrenia spectrum disorder were recruited via the French national network of schizophrenia Expert Centres. Exclusion criteria included ongoing acute psychotic episode or the occurrence of hospitalisation or treatment change within the 4 weeks before assessment. Participants were invited to complete a thorough assessment protocol over one or two visits, where they provided demographic and clinical information, filled out standardised questionnaires, completed neuropsychological assessments and underwent physical examinations and bloodwork. The collected data were used to make personalised therapeutic recommendations, as well as stored anonymously in a shared online platform (e-schizo©) for research purposes. The overall protocol received approval from the relevant ethical and data protection bodies (respectively, CPP-Ile-de-France IX, January 18, 2010, and CNIL).

### 2.2 Variables

#### 2.2.1 Childhood trauma

The CTQ is a 25-item retrospective, self-report questionnaire used to assess childhood maltreatment early in life (Bernstein et al., 1997; Paquette et al., 2004). The CTQ distinguishes five different subtypes of trauma – emotional abuse (EA), physical abuse (PA), sexual abuse (SA), emotional neglect (EN), and physical neglect (PN). *Every item is rated on a 5-point Likert-type scale (1 = never true, 5 = Very often true) and allow for computation of both mean scores for each type of trauma (range 5-25) and categorical scores, with fixed threshold values for each subdomain (≥13 for emotional abuse; ≥10 for physical abuse; ≥8 for sexual abuse; ≥15 for emotional neglect and ≥10 for physical neglect) (*Bernstein et al., 1997; Paquette et al., 2004*)*. *We used CTQ scores as continuous variables to enhance statistical sensitivity in assessing associations with other continuous clinical or biological measures.*

#### 2.2.2 Inflammation

The four studied inflammatory parameters were selected based on the literature on inflammation as related to CT: IL-1β, IL-6, TNF-α and CRP (Baumeister et al., 2016; Coelho et al., 2014; Danese et al., 2007; Ermakov et al., 2022; Fernandes et al., 2016; Goldsmith et al., 2016). *Venous blood was obtained from subjects without requiring fasting between 7:00 a.m. and 9:00 a.m. on weekdays. Five milliliters of peripheral blood were drawn by venipuncture and allowed to clot for 1 h before centrifugation. Serum samples were stored in 0.*5 ml *aliquots at −80 °C and thawed on ice at the time of analysis. The concentrations of IL-1β, IL-6, TNF-α, and CRP were measured using electrochemiluminescence-based multiplex assays (Meso Scale Discovery, Rockville, Maryland, USA). All assays were performed according to the manufacturer's instructions. Each sample was measured in singlicate. Intra-assay variability was assessed using duplicate measurements of quality control samples at high, medium, and low concentrations provided by the kit, and included on each plate. Recovery rates for each QC point were calculated for each cytokine and found to be within acceptable limits (80 %–120 % of expected concentrations). To preserve sample integrity, the number of freeze-thaw cycles was strictly controlled and did not exceed three.*

#### 2.2.3 Clinical dimensions

The PANSS is a clinical tool used to assess schizophrenia symptoms (Kay et al., 1987; Lançon et al., 1999). Each of the 30 items was scored using a 7-point Likert rating system. Symptoms on the PANSS cluster under five distinct factors– positive (Pos.), negative (Neg.), disorganised (Dis.), excited (Exc.) and depressed (Dep.) (Wallwork et al., 2012). *The five-factor model of the PANSS provides a more nuanced and clinically relevant assessment of symptom dimensions in schizophrenia compared to the traditional three-subscale or total score approaches. It reflects the underlying heterogeneity of schizophrenia symptoms more accurately, Emerging evidence suggests that specific biological markers (*e.g.*, inflammation, neuroimaging findings) are more strongly associated with individual symptom dimensions rather than global scores* (Liu et al., 2024; Zhou et al., 2024*)*. *The five-factor model thus enables more targeted and biologically meaningful analyses.*

### 2.3 Statistical analyses

Following guidance from prior literature, inflammatory parameters’ data entries corresponding to concentrations under or above the limits of detection (LOD) for that biomarker, as determined by the assay manufacturer, were imputed with, respectively, the lower limit of detection (LLOD)/2, and with the maximum value registered for that biomarker + 1. If more than 50 % of the measured concentrations for a particular biomarker fell outside its limits of detection, it was removed from further analyses (Khalfallah et al., 2021; Leboyer et al., 2021). R software was used and missing data were imputed with the “VIM” package, using a K-nearest neighbour approach (Kowarik and Templ, 2016).

Path analysis is a statistical method adjacent to structural equation modelling used to evaluate temporal relationships between sets of variables. It is able to simultaneously assess overall model fit to the data and test all the associations between each individual variable. The path analysis used a maximum likelihood parameter estimator (MLR) resistant to non-normal distribution (Liang et al., 2019; Muthén and Muthén, 2007). The final goodness of fit of the model was assessed by several methods set at the following thresholds: a non-significant (p-value >0.05) Chi-Square test; a Root Mean Square Error of Approximation (RMSEA) value of 0.05 or less; a Comparative Fit Index (CFI) of greater than 0.90; a Tucker-Lewis Index (TLI) value of 0.95 or higher. A secondary model including adjustment for possible confounding factors - age, sex, body mass index (BMI) and dichotomous cigarette smoking status - was also produced. Path analyses were run on the MPlus software.

We have also investigated, one by one, the relationships between each childhood trauma subtype and each inflammatory parameter, and in turn between each inflammatory parameter and each symptom dimension. First, univariate relationships between the childhood trauma scores and concentrations of the selected inflammatory parameters, and between the latter and symptom dimensions scores, were investigated. Given their non-normal distribution, Spearman rank correlation tests were used. If a significant association (p-value<0.05) was found at this stage, it was further tested at the multivariable level with multiple linear regression.

## 3 Results

The sample consisted of 451 subjects with a mean age of 30.4 years (SD = 8.53) and a male-to-female ratio of approximately 3:1. *The vast majority of participants (79.16 %) reported at least on type of trauma on the CTQ, with emotional neglect being the most common trauma type, reported by 62.75 % of participants, followed respectively by emotional abuse (47.90 %), physical neglect (41.02 %), sexual abuse (23.06 %) and physical abuse (18.85 %). The total PANSS score of the sample can be considered as moderate. The profile of the sample corresponds to subjects with few positive symptoms and excitement, but a clinical presentation marked mainly by negative symptoms and, to a lesser extent, moderate cognitive disorganization. There is also a mild depressive/anxious component*. More detailed descriptive variables, as well as the mean and standard-deviations of the CTQ and PANSS scores are presented in Table 1.

**Table 1:** Sample characteristics in terms of the variables of interest; N and % are presented for categorical variables and mean and standard deviation for continuous variables; n = 451.

	N (%)/Mean (SD)
Sex (women)	114 (25.28 %)
Age	30.44 (8.53)

Body mass index (BMI)	26.98 (5.72)
Current smoker (yes)	235 (52.11 %)

CTQ scores:	
Emotional abuse (EA)	9.56 (4.38)
Physical abuse (PA)	6.38 (2.62)
Sexual abuse (SA)	6.19 (3.03)
Emotional neglect (EN)	11.57 (4.2)
Physical neglect (PN)	7.63 (2.55)
Total	41.31 (11.86)

PANSS scores:	
Positive factor (Pos.)	8.71 (4.33)
Negative factor (Neg.)	15.87 (6.73)
Disorganised factor (Dis.)	7.49 (3.76)
Excited factor (Exc.)	5.32 (2.14)
Depressed factor (Dep.)	7.15 (3.25)
Total	65.38 (20.10)

The inflammatory variables are described in Table 2. Due to the high percentage of inputs corresponding to concentrations falling outside the limits of detection, IL-1β was removed from subsequent statistical analyses.

**Table 2:** Inflammatory biomarkers; namely, parameters used for data treatment and descriptive statistics for serum concentrations (in pg/mL) of each selected biomarker in the included FACE-SZ sample; n = 451.

	Kit LOD range (LLOD – ULOD)	% outside LOD	Mean	Range (min-max)
IL1-β	0.05–375	66.07^+^	1.36	0.02–431.19
IL-6	0.06–488	8.20	2.14	0.03–123.14
TNF-α	0.44–248	0	4.73	0.31–141.50
CRP	1.33–49,600	0.88	4320000	0.66–76,000,000

Exceeds threshold of 50 % of measurements falling outside the LOD range.

There were no significant correlations between any of the childhood trauma scores (total and subtypes) and serum concentrations of the selected inflammatory parameters. Regarding the association between the inflammatory biomarkers and symptom dimensions, there was a significant positive correlation between TNF-α and the PANSS negative factor (ρ = 0.12, p-value = 0.01), as well as between CRP and the positive (ρ = 0.12, p-value = 0.01), negative (ρ = 0.09, p-value = 0.05), and disorganised factors (ρ = 0.12, p-value = 0.01) and with the PANSS total score (ρ = 0.10, p-value = 0.04) (Supplementary Table 1S). The significant associations found at the univariate level were subsequently explored with regression analyses adjusting for age, sex, BMI and cigarette smoking. Five different regressions were run – one with TNF-α as the explanatory variable and PANSS negative score as the response variable, and the next ones with CRP as the explanatory variable and PANSS positive, negative, disorganised or total scores as the response variables. Of these, only the CRP-positive symptoms association remained significant (β = 0.07, p = 0.04) (Supplementary Table 2S).

**Fig. 1:**
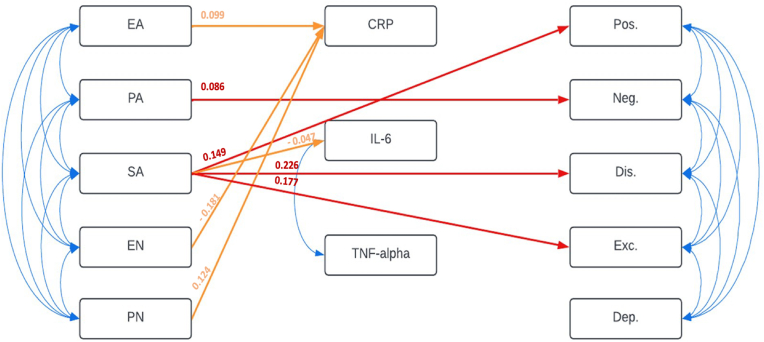
Path-analysis diagram modelling the relationships between CT types, selected peripheral inflammatory biomarkers, and PANSS factors. Legends: Arrows imply significant associations (p-value<0.05); the colour blue represents correlations, while red and orange arrows (paths) depict direct and indirect effects, respectively; the given values correspond to the standardised regression coefficients for each path Abbreviations: Dep: depressed PANSS factor, Dis.: disorganised PANSS factor, EA: emotional abuse, EN: emotional neglect, Exc.: excited PANSS factor, Neg.: negative PANSS factor, PA: physical PANSS abuse, PN: physical neglect, Pos.: positive PANSS factor, SA: sexual abuse.

The path analysis model displayed good fit to the data (Chi-squared = 40.39, d.f. = 39, p-value = 0.83; RMSEA = 0.01, p-value = 1.00; CFI = 1.00; TLI = 1.00**)**. Scores of the five CT subtypes were correlated with each other, as were the different PANSS factors. The two cytokines, IL-6 and TNF-α, were correlated with each other, while neither were with CRP. There were significant direct effects of given CTQ subtypes on specific PANSS dimensions. Namely, there were positive associations between physical abuse and negative symptoms (β = 0.09, p = 0.03) and between sexual abuse and positive (β = 0.15, p < 0.01), disorganised (β = 0.23, p < 0.01) and excited symptoms (β = 0.18, p = 0.02). With regards to CT and the inflammatory biomarkers, levels of CRP were significantly predicted by scores of emotional abuse (β = 0.10, p = 0.01) and physical neglect (β = 0.12, p < 0.01). There was a negative association between CRP and emotional neglect (β = −0.18, p < 0.01), and between IL-6 and sexual abuse (β = −0.05, p < 0.01). TNF-α levels were not significantly predicted by any of the CTQ scales. *In the path-analysis, t*here were no significant links between levels of CRP, IL-6, TNF-α and the PANSS factors. Therefore, no mediation effect of these biomarkers on the relationship between CT types and symptoms dimensions was observed. *The results of the path-analysis are available in* Fig. 1. *Adjustment for the possible confounders (i.e. age, sex, body mass index and tobacco smoking status) in a separate path-analysis model, equally significant according to the model fit indices, did not change the results described above* (Supplementary Fig. 1).

## 4 Discussion

Our path-analysis of a large cohort of subjects with schizophrenia corroborated prior findings linking CT types to specific PANSS dimensions and identified significant novel associations between CT and selected inflammatory biomarkers. As found in the literature and in prior works with the FACE-SZ cohort, there were positive direct associations between physical abuse and negative symptoms and between sexual abuse and positive, disorganised and excited dimensions (Baudin et al., 2016; Pignon et al., 2019; Sitko et al., 2014). Regarding associations between CT and inflammatory biomarkers, the CRP levels were positively predicted by emotional abuse and physical neglect and negatively by emotional neglect, and IL-6 levels were negatively predicted by sexual abuse. Given the absence of significant associations between these inflammatory parameters and the PANSS factors scores, a mediatory role for these biomarkers in the CT-clinical dimensions relationship could not be determined. The positive relationship between CT, namely emotional abuse and physical neglect, and CRP, a broad indicator of chronic peripheral inflammation, is in accordance with several studies (Baumeister et al., 2016; Brown et al., 2021; Coelho et al., 2014; Dennison et al., 2012). While the precise biological mechanisms underpinning the effect of CT on chronic inflammation are unknown, there is evidence for the implication of the innate immune response, as shown by the exacerbated cytokine response following ex-vivo bacterial lipopolysaccharide (LPS) stimulation in individuals with a history of severe CT, particularly emotional abuse (de Koning et al., 2022).

Conversely, the negative associations between emotional neglect and CRP and between sexual abuse and IL-6 were unanticipated and mechanistically counter-intuitive in light of extensive research demonstrating a link between stress and increased pro-inflammatory markers. One line of explanation for this finding could be related to the complex role of IL-6 and, downstream, of CRP in the regulation of the immune response. While it is generally recognised as a pro-inflammatory cytokine, IL-6 is a signalling protein with a wide and complex range of effects, including anti-inflammatory mechanisms, which may posit that low levels of it, as well as high levels, may be associated with chronic inflammation (Scheller et al., 2011). As CRP production by the liver is stimulated by IL-6, lower levels of this cytokine would conceivably be associated with decreased CRP as well. Similarly to IL-6, anti-inflammatory effects of CRP have been described (Marnell et al., 2005). Alternatively, as both CT and psychosis are independently associated with elevations in IL-6 and CRP, it can be hypothesized that their overexpression may favour a negative feedback effect with consequent reduction of their production. This might be especially relevant according to the delay time between the psychological trauma, first symptoms and the moment of study assessment during which compensatory biological processes counteracting inflammation may have taken place. Yet, it must be critically stated that the above explanations represent theoretical hypotheses only, and that the results of the present study must be replicated before further investigation.

This study was the first to directly test the long-hypothesized mediation effect of inflammatory biomarkers using a model which encompassed CT, inflammatory biomarkers, and clinical measures (Danese and Baldwin, 2017). A major strength of this study was the use of a large sample of clinically and biologically well-characterised subjects. Furthermore, this project innovatively applied path analysis within the immuno-psychiatry field, an efficient and refined approach to modelling multivariable relationships. Still, a few limitations must be considered. *Childhood trauma was reported retrospectively, with the inherent weakness of the retrospective design. These measures of childhood trauma may be affected by recall bias and cognitive impairments or positive symptoms associated with psychotic disorder (*Baldwin et al., 2019; Bernstein et al., 1997; Hardt and Rutter, 2004*)*. Second were elements related to the inflammatory parameters data, namely the exclusion of IL-1β due to the amount of abnormal concentration readings for this cytokine. Additionally, given the limited information on the interaction between trauma-associated and schizophrenia immune profiles, possibly relevant biomarkers may have flown under the radar. *Although the selection of these specific biomarkers is well-rooted in prior evidence, a suggestion could be made for the use of composite inflammatory indices, incorporating a wider range of interrelated inflammatory variables, in future studies. An argument could also be made for the use of multivariable inflammatory indexes rather than specific biomarkers* (de Koning et al., 2022; Khalfallah et al., 2021*)*. *Third and finally, antipsychotic treatments were not accounted for as potential confounding variable in the path-analysis despite conflicting evidence regarding their effect on inflammation (*Ermakov et al., 2022; Fernandes et al., 2016*)*. *Modeling their effects in statistical analyses is challenging, particularly in the absence of detailed data on treatment type, dosage, and duration. Moreover, adjusting for BMI effectively accounts for the primary pathway through which antipsychotics influence inflammation, namely weight gain*.

In conclusion, the results from our path analysis support the existence of associations between CT and inflammation within schizophrenia. It is also possible that the inflammatory parameters studied are associated with an increased risk of schizophrenia but not with its phenotypic expression (*i.e.*, as a risk factor but not a modifier factor).

Elucidating the relationship between these three elements represents an important step in understanding the biological mechanisms linking environmental influences to clinical symptoms of schizophrenia, and potentially point towards novel biomarkers and treatment targets for a particular phenotype rooted in CT and chronic inflammation (Ermakov et al., 2022). The present methodology could also be used to model the effect of other early-life environmental factors, such as obstetric complications, on inflammation and schizophrenia. Alternatively, it could be pertinent to look at the impact of these factors on other clinical measures besides the PANSS, such as cognitive outcomes, which are also impacted by immune dysregulations (Bulzacka et al., 2016; Patlola et al., 2023). Ultimately, further research in immuno-psychiatry could help identify distinct disorder phenotypes, opening new avenues for patient stratification, treatment, and diagnosis.

## CRediT authorship contribution statement

**Francisca Silva:** Writing – original draft, Methodology, Conceptualization. **Andrei Szöke:** Writing – review & editing, Validation, Supervision, Methodology, Data curation. **Mohamed Lajnef:** Writing – review & editing, Methodology, Formal analysis. **Myrtille André:** Writing – review & editing, Data curation. **Christelle Andrieu:** Writing – review & editing, Data curation. **Jihene Bouassida:** Writing – review & editing, Data curation. **Wahid Boukouaci:** Writing – review & editing, Data curation. **Fabrice Berna:** Writing – review & editing, Data curation. **Delphine Capdevielle:** Writing – review & editing, Data curation. **Maud Cléry:** Writing – review & editing, Data curation. **Isabelle Chéreau:** Writing – review & editing, Data curation. **Nathalie Coulon:** Writing – review & editing, Data curation. **Julia Clauss-Kobayashi:** Writing – review & editing, Data curation. **Eric Fakra:** Writing – review & editing, Data curation. **Jean-Michel Dorey:** Writing – review & editing, Data curation. **Caroline Dubertret:** Writing – review & editing. **Guillaume Fond:** Writing – review & editing. **Ophélia Godin:** Writing – review & editing, Methodology. **Tudi Goze:** Writing – review & editing, Data curation. **Marion Leboyer:** Writing – review & editing. **Sylvain Leignier:** Writing – review & editing, Data curation. **Pierre-Michel Llorca:** Writing – review & editing, Data curation. **Jasmina Mallet:** Writing – review & editing, Data curation. **David Misdrahi:** Writing – review & editing, Data curation. **Nicolas Oriol:** Writing – review & editing, Data curation. **Romain Rey:** Writing – review & editing, Data curation. **Paul Roux:** Writing – review & editing. **Benoit Schorr:** Writing – review & editing, Data curation. **Arnaud Tessier:** Writing – review & editing, Data curation. **Ryad Tamouza:** Writing – review & editing, Supervision, Methodology, Data curation. **Mathieu Urbach:** Writing – review & editing, Data curation. **Etienne Véry:** Writing – review & editing, Data curation. **Baptiste Pignon:** Writing – review & editing, Methodology, Data curation, Conceptualization. **Franck Schürhoff:** Writing – review & editing, Data curation.

## Declaration of competing interest

The authors declare no conflicts of interest relevant to this work.

## Data Availability

The data that has been used is confidential.
