# Supplementary material for: Inflammation, childhood trauma, and symptom dimensions in schizophrenia: a path-analysis study
Source: Brain Behav Immun Health. 2025 Jul 16;48:101060. doi: 10.1016/j.bbih.2025.101060 (PMC12490581; doi:10.1016/j.bbih.2025.101060)
Supplement: Multimedia component 1 [file mmc1.docx]

| *Table 1S: Spearman correlations between inflammatory parameters and CTQ and PANSS scores* | | | | | | | | | | | | | | | | | |
| --- | --- | --- | --- | --- | --- | --- | --- | --- | --- | --- | --- | --- | --- | --- | --- | --- | --- |
|  | **EA** | |  | **PA** | |  | **SA** | |  | **EN** | |  | **PN** | |  | **Total** | |
|  | ρ | (p) |  | ρ | (p) |  | ρ | (p) |  | ρ | (p) |  | ρ | (p) |  | ρ | (p) |
| IL-6 | 0.06 | (0.20) |  | -0.02 | (0.62) |  | 0.01 | (0.88) |  | -0.04 | (0.43) |  | 0.05 | (0.25) |  | 0.01 | (0.90) |
| TNF-α | 0.04 | (0.39) |  | -0.04 | (0.43) |  | 0.00 | (0.97) |  | 0.07 | (0.16) |  | 0.03 | (0.49) |  | 0.08 | (0.08) |
| CRP | 0.07 | (0.12) |  | 0.02 | (0.61) |  | -0.03 | (0.49) |  | -0.05 | (0.25) |  | 0.04 | (0.42) |  | 0.05 | (0.31) |
|  |  |  |  |  |  |  |  |  |  |  |  |  |  |  |  |  |  |
|  | **Pos.** | |  | **Neg.** | |  | **Dis.** | |  | **Exc.** | |  | **Dep.** | |  | **Total** | |
|  | ρ | (p) |  | ρ | (p) |  | ρ | (p) |  | ρ | (p) |  | ρ | (p) |  | ρ | (p) |
| IL-6 | 0.03 | (0.46) |  | 0.03 | (0.52) |  | 0.03 | (0.52) |  | -0.02 | (0.67) |  | 0.06 | (0.21) |  | 0.02 | (0.70) |
| TNF-α | 0.03 | (0.49) |  | **0.12** | **(0.01)*** |  | 0.03 | (0.51) |  | 0.00 | (0.99) |  | 0.08 | (0.09) |  | 0.07 | (0.17) |
| CRP | **0.12** | **(0.01)*** |  | **0.09** | **(0.05)*** |  | **0.12** | **(0.01)*** |  | 0.01 | (0.89) |  | 0.07 | (0.11) |  | **0.10** | **(0.04)*** |
|  |  |  |  |  |  |  |  |  |  |  |  |  |  |  |  |  |  |
| **Significant p-value, at the 0.05 threshold* | | | | | | | | | | | | | | | | | |
| ρ=Spearman rank coefficient; (p)=p-value | | | | | | | | | | | | | | | | | |

| *Table 2S: significant inflammation-PANSS associations at the multivariable level; regression coefficients (β) and corresponding p-values for each variable in the five different multiple regression models, with the predictor and response variable of interest in bold* | | | | | |
| --- | --- | --- | --- | --- | --- |
| Response: PANSS Neg. | | | | | |
| Explanatory: | **TNF-α** | Age | Sex | BMI | Smoking |
| β | 0.01 | -0.05 | 2.24 | 0.05 | 0.06 |
| (p) | (0.76) | (0.20) | (<0.01)* | (0.39) | (0.92) |
|  |  |  |  |  |  |
| Response: PANSS Pos. | | | | | |
| Explanatory: | **CRP** | Age | Sex | BMI | Smoking |
| β | **0.07** | 0.01 | -0.21 | 0.03 | 0.29 |
| (p) | **(0.04)*** | (0.61) | (0.66) | (0.41) | (0.49) |
|  |  |  |  |  |  |
| Response: PANSS Neg. | | | | | |
| Explanatory: | **CRP** | Age | Sex | BMI | Smoking |
| β | 0.03 | -0.05 | 2.27 | 0.04 | 0.00 |
| (p) | (0.56) | (0.18) | (<0.01)* | (0.50) | (1.00) |
|  |  |  |  |  |  |
| Response: PANSS Dis. | | | | | |
| Explanatory: | **CRP** | Age | Sex | BMI | Smoking |
| β | 0.03 | 0.02 | 0.38 | 0.00 | 0.30 |
| (p) | (0.36) | (0.37) | (0.36) | (0.99) | (0.41) |
|  |  |  |  |  |  |
| Response: PANSS Total | | | | | |
| Explanatory: | **CRP** | Age | Sex | BMI | Smoking |
| β | 0.15 | 0.09 | 4.10 | 0.03 | 0.27 |
| (p) | (0.31) | (0.40) | (0.06) | (0.85) | (0.88) |
|  | | | | | |
| **Significant p-value, at the 0.05 threshold* | | | | | |
| β=regression coefficient; (p)=p-value | | | | | |
