# Supplementary material for: Inflammation, childhood trauma, and symptom dimensions in schizophrenia: a path-analysis study
Source: Brain Behav Immun Health. 2025 Jul 16;48:101060. doi: 10.1016/j.bbih.2025.101060 (PMC12490581; doi:10.1016/j.bbih.2025.101060)
Supplement: Multimedia component 2 [file mmc2.pptx]

## Slide 1
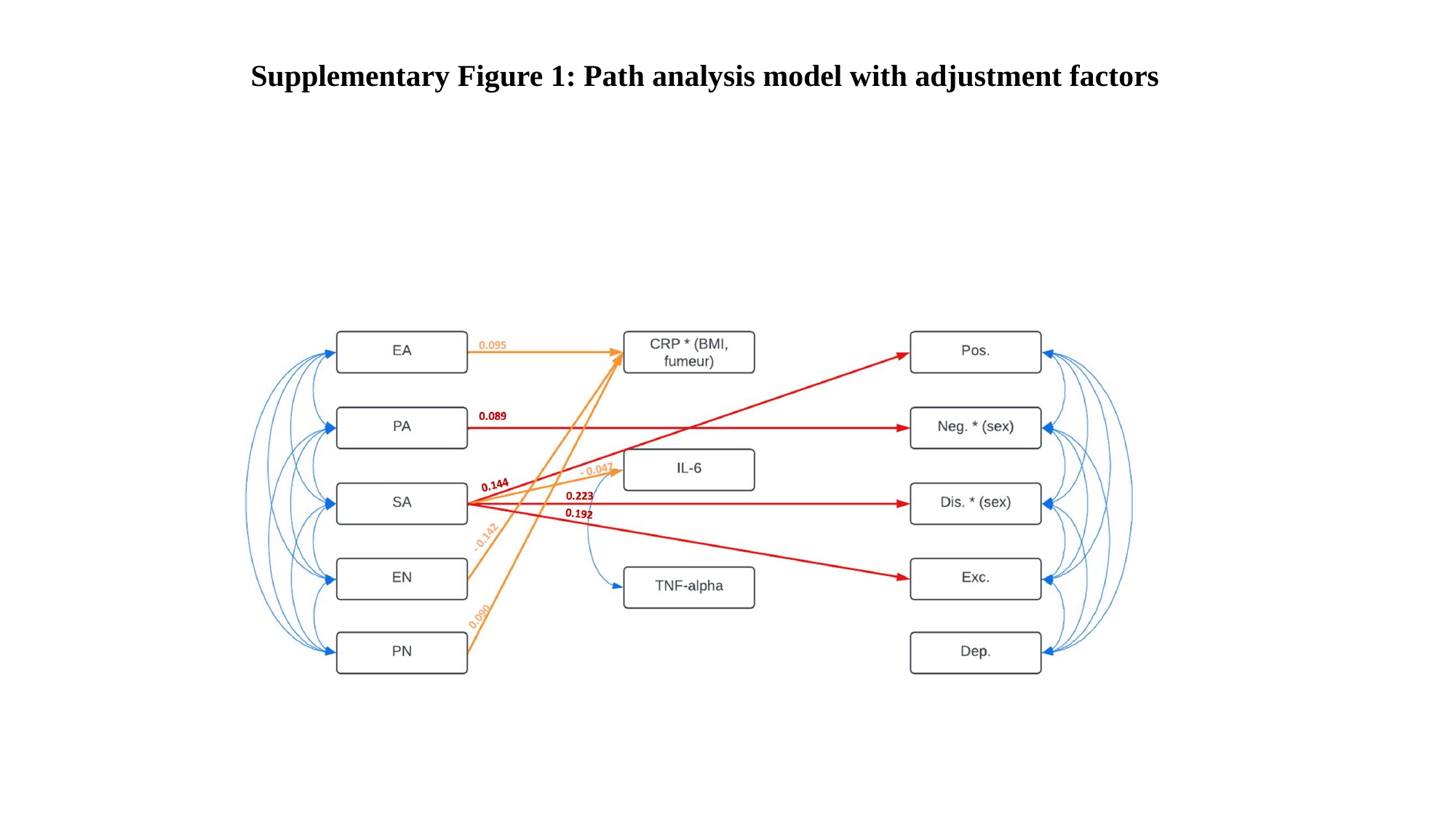

Supplementary Figure 1: Path analysis model with adjustment factors
Figure I: path analysis diagram modelling the relationships between CT types, selected peripheral inflammatory biomarkers, and PANSS factors, adjusted for possible confounding factors; arrows imply significant associations (p-value<0.05); the colour blue represents correlations, while red and orange arrows (paths) depict direct and indirect effects, respectively; the given values correspond to the estimated regression coefficients for each path; asterisks inside the boxes indicate the adjustment factors which also significantly predicted that variable; model fit indices: Chi-squared=126.86, d.f.=72, p-value=0.91; RMSEA=0.04, p-value=0.89; CFI=0.91; TLI=0.89
